# Vpu Downmodulates Two Distinct Targets, Tetherin and Gibbon Ape Leukemia Virus Envelope, through Shared Features in the Vpu Cytoplasmic Tail

**DOI:** 10.1371/journal.pone.0051741

**Published:** 2012-12-19

**Authors:** Tiffany M. Lucas, Sanath K. Janaka, Edward B. Stephens, Marc C. Johnson

**Affiliations:** 1 Department of Molecular Microbiology and Immunology, Christopher S. Bond Life Science Center, University of Missouri-School of Medicine, Columbia, Missouri, United States of America; 2 Department of Microbiology, Molecular Genetics and Immunology, University of Kansas Medical Center, Kansas City, Kansas, United States of America; Lady Davis Institute for Medical Research, Canada

## Abstract

During human immunodeficiency virus-1 (HIV-1) assembly, the host proteins CD4 (the HIV-1 receptor) and tetherin (an interferon stimulated anti-viral protein) both reduce viral fitness. The HIV-1 accessory gene Vpu counteracts both of these proteins, but it is thought to do so through two distinct mechanisms. Modulation of CD4 likely occurs through proteasomal degradation from the endoplasmic reticulum. The exact mechanism of tetherin modulation is less clear, with possible roles for degradation and alteration of protein transport to the plasma membrane. Most investigations of Vpu function have used different assays for CD4 and tetherin. In addition, many of these investigations used exogenously expressed Vpu, which could result in variable expression levels. Thus, few studies have investigated these two Vpu functions in parallel assays, making direct comparisons difficult. Here, we present results from a rapid assay used to simultaneously investigate Vpu-targeting of both tetherin and a viral glycoprotein, gibbon ape leukemia virus envelope (GaLV Env). We previously reported that Vpu modulates GaLV Env and prevents its incorporation into HIV-1 particles through a recognition motif similar to that found in CD4. Using this assay, we performed a comprehensive mutagenic scan of Vpu in its native proviral context to identify features required for both types of activity. We observed considerable overlap in the Vpu sequences required to modulate tetherin and GaLV Env. We found that features in the cytoplasmic tail of Vpu, specifically within the cytoplasmic tail hinge region, were required for modulation of both tetherin and GaLV Env. Interestingly, these same regions features have been determined to be critical for CD4 downmodulation. We also observed a role for the transmembrane domain in the restriction of tetherin, as previously reported, but not of GaLV Env. We propose that Vpu may target both proteins in a mechanistically similar manner, albeit in different cellular locations.

## Introduction

Vpu is an 81–86 amino acid, type-1 transmembrane protein found in HIV-1 and a few closely related strains of SIV. Vpu modulates a wide range of targets including the host proteins CD4, tetherin, IkB, MHC-II, NTB-A, and the gammaretroviral gibbon ape leukemia virus (GaLV) envelope (Env) [1]–[9]. Of these functions, Vpu's ability to modulate cellular CD4 and tetherin (BST-2, CD137) have been the best described [10]–[13]. CD4 is the primary receptor for HIV-1. Vpu targets newly synthesized CD4 in the rough endoplasmic reticulum (RER) through interactions between the cytoplasmic tails (CT) of Vpu and CD4, recruiting the Skp1-Cullin-β-TrCP E3-ubiquitin ligase complex, resulting in the subsequent proteasomal degradation of CD4 [5], [14]–[19]. The cytoplasmic tail (CT) of Vpu is unambiguously required for CD4 modulation, but it is disputed whether the membrane spanning domain (MSD) also plays a specific role [20]–[25].

Tetherin is an interferon inducible, type-II transmembrane anti-viral protein with a C-terminal GPI-anchor. Tetherin, as its name suggests, “tethers” many budding enveloped viruses or virus like particles to the plasma membrane (PM), including retroviruses, Ebola, Kaposi sarcoma-associated herpes virus (KSHV) and influenza virus like particles [9], [26]–[28]. Vpu-mediated antagonism of tetherin requires an interaction between the MSDs of Vpu and tetherin, but as of yet, there is no consensus on the precise mechanism by which Vpu modulates tetherin activity. Vpu has been reported to reduce tetherin surface expression by altering the rate of recycled and/or restricting newly synthesized tetherin from reaching the PM [29]–[34]. However, it has also been reported that Vpu can modulate tetherin activity in the absence of surface downmodulation and intracellular depletion [35]. Some studies suggest that tetherin can be degraded through β-TrCP mediated targeting to lysosomes or the proteasome [33], [36], [37].

Although the mechanisms for CD4 and tetherin antagonism are believed to be distinct, evidence suggests that Vpu contains some shared features required for modulation of both proteins. For instance, complete proscription of either target requires two critical serines housed in the Vpu cytoplasmic tail, which is also required for interaction with β-TrCP and degradation of tetherin or CD4 [17], [37], [38]. Vpu mutants lacking these serine residues retain some activity against tetherin but not CD4 [34], [39]. Direct parallels between Vpu modulation of tetherin and CD4 are difficult to draw due to differences and limitations in the assays employed. Studies investigating tetherin antagonism have relied heavily on detection of viral particle release, through protein release or infectious virus production, although some studies have also measured tetherin modulation directly. Reports on CD4 down-modulation typically rely on biochemical assays measuring total protein or surface expression. Additionally, Vpu studies have used different cell types, multiple methods of introducing CD4 or tetherin targets (endogenous or exogenous), and different methods of producing Vpu (e.g., native or codon-optimized, contained in the provirus or introduced *in trans*). Employment of these disparate protocols limits the ability to directly compare different studies.

We and others found that Vpu prevents GaLV Env incorporation into HIV-1 particles, likely through a shared structural recognition motif INxxIxxVKxxVxRxK in the Env cytoplasmic tail that resembles the critical Vpu sensitivity motif found in the cytoplasmic tail of CD4 [1]–[3]. This motif is conserved and is transferrable to confer sensitivity in previously insensitive proteins [1]. Based on these findings, we currently believe Vpu mistakenly recognizes the cytoplasmic tail of GaLV Env as a CD4 analogue. Similarly, GaLV Env is packaged into the virus in the absence of Vpu, however, unlike CD4, GaLV Env can form infectious pseudotyped virus to assess incorporation of the target protein. Modulation of GaLV Env by Vpu is sensitive and well suited for a comparative study with the modulation of tetherin by Vpu. By employing GaLV Env, constraint of both distinct targets can be studied in the same cell type using Vpu encoded in the provirus with infectivity as the output for both.

## Materials and Methods

### Cell lines

The human endothelial kidney (HEK) 293FT, 293 mCAT-1, and 293 TVA [40] cells were obtained from Invitrogen, W. Mothes, and J. Young, respectively. All cells were cultured in DMEM supplemented with 10% fetal bovine serum, 2 mM L-glutamine, 1 mM sodium pyruvate, and 10 mM non-essential amino acids.

### Plasmids

The NL4-3 derived HIV-CMV-GFP was kindly provided by Vineet KewalRamani. This clade B provirus lacks Env, Vpr, Vpu, Nef, and Vif, and contains a green fluorescent protein (GFP) gene in place of the Nef gene under the control of a cytomegalovirus (CMV) promoter. The gene expressing the far red fluorescent protein E2Crimson was engineered into this construct to exactly replace the GFP gene to produce HIV-CMV-E2Crimson. A library of HXB2 Vpu mutants was derived from a previously described HXB2 parent Vpu [22]. Mutations to the Vpu gene were generated either by linker insertion, or by two-step PCR, and the genes were subsequently subcloned into the unique *Nhe*I and *Asc*I sites, which were engineered immediately upstream and downstream of the natural location of the Vpu gene in HIV-CMV-E2Crimson. The human tetherin expression construct tetherin-HA [41] was kindly provided by P. Bieniasz. The vesicular stomatitis virus glycoprotein (VSV-G), murine leukemia virus (MLV)/GaLV Env, and Rous sarcoma virus (RSV) Env ΔCT expression constructs have been described previously [2], [42].

### Infectivity analysis

293FT cells were plated in 6-well plates and allowed to reach 60% confluency prior to transfection. For tetherin studies, 293FT cells were transfected with the following expression constructs: provirus (425 ng) and VSV-G (25 ng) with or without 12.5 ng of tetherin-HA in a total of 500 ng. For GaLV Env assays, cells received 500 ng provirus, 25 ng of RSV Env ΔCT, and 475 ng MLV/GaLV Env (GaLV Env) [1], [2]. Plasmids were transfected with polyethylenimine (PEI) at a concentration of 1 ug DNA per 4 ul (1 mg/1 ml stock concentration) and media was replaced 6 hours later. At 48 h post-transfection, media containing virus was collected and frozen overnight. For tetherin studies, the media was used to transduce 293T mCAT-1 cells expressing the murine leukemia virus Env receptor (mCAT-1). For GaLV Env studies, media was used in parallel to transduce both 293 mCAT-1 cells and 293T TVA, which expresses the RSV receptor (TVA). Two days later, cells were collected, fixed with 4% paraformaldehyde and analyzed by flow cytometry using an Accuri flow cytometer. Cells transduced by virus were gated by E2Crimson expression in the FL4 channel to determine viral infectivity.

## Results

**Figure 1:**
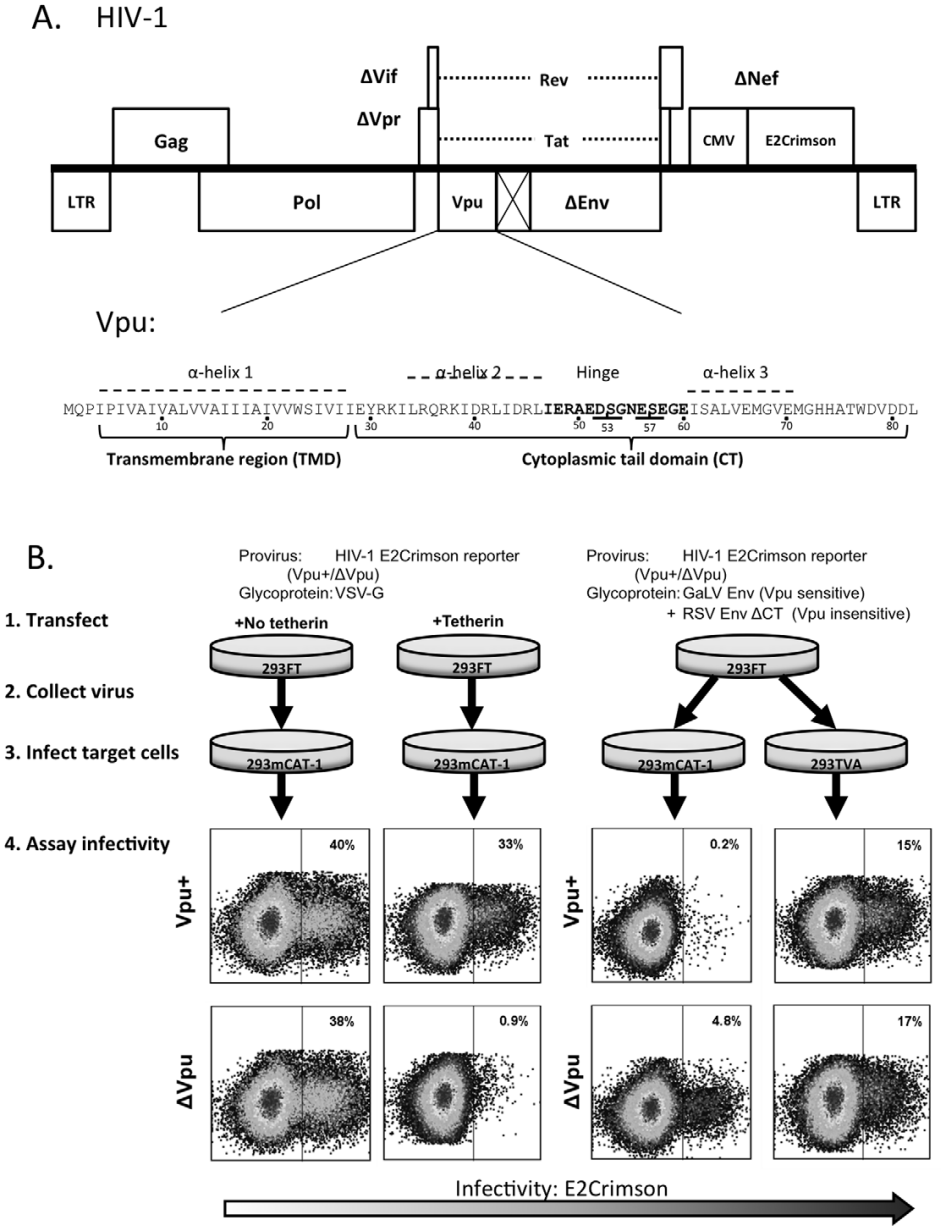
Schematics of HIV-1 proviral construct and experimental assay. (A) HIV-1 NL4-3 proviral construct with E2Crimson reporter showing enlargement of Vpu schematic outlining critical features in Vpu. Dotted outline predicted α-helices [22], bold script indicates the hinge region and underlined script highlights phosphorylated serines at positions 53,57. (B) For tetherin assays, 293FT cells were transfected with the following expression constructs: provirus and VSV-G with or without tetherin. For GaLV Env assays, cells received provirus, RSV Env ΔCT, and MLV/GaLV Env (GaLV Env) [1], [2]. Transduced cells were analyzed by flow cytometry two days later. Flow plots illustrate typical data output for positive controls (X-axis: E2Crimson expression, Y-axis: SSC).

To determine specific regions of Vpu mediating antagonism of tetherin and GaLV Env, we generated a library of HXB2 Vpu mutants and introduced them into a reduced HIV-1 clade B proviral construct containing an E2Crimson reporter gene (Figure 1A). HXB2 Vpu was used, as several mutants had previously been generated [22]. For tetherin modulation assays, each provirus was transfected with a VSV-G expression plasmid alone, or in combination with an HA-tagged tetherin expression construct [41] (Figure 1B, left). Vpu activity was measured by comparing infectivity in the presence and absence of tetherin. For assaying GaLV Env modulation, an internally controlled system was used where each mutant was transfected with a mixture of plasmids expressing the previously described Vpu-sensitive chimeric MLV Env containing the GaLV Env cytoplasmic tail, herein referred to simply as GaLV Env, and a Vpu-insensitive RSV Env lacking the cytoplasmic tail (RSV Env ΔCT) (Figure 1B, right). Virus was collected and used to transduce 293T mCAT-1, expressing the MLV Env receptor (mCAT-1) and 293T TVA, which expresses the RSV receptor (TVA). Vpu activity was measured by comparing the ratio of RSV Env pseudotyped infectious virus to MLV Env pseudotyped infectious virus. For both assays, infections were quantified by flow cytometry, and activity was expressed by normalizing to a provirus with wildtype Vpu (Vpu wt) (100% activity) and a Vpu-deficient provirus (ΔVpu) (0%). It should be noted that the raw output is inverted between the two assays: with tetherin, Vpu enhances infectivity, but with GaLV Env, Vpu inhibits infectivity.

### Restriction is highly dependent on Vpu cytoplasmic tail, but not transmembrane region

**Figure 2:**
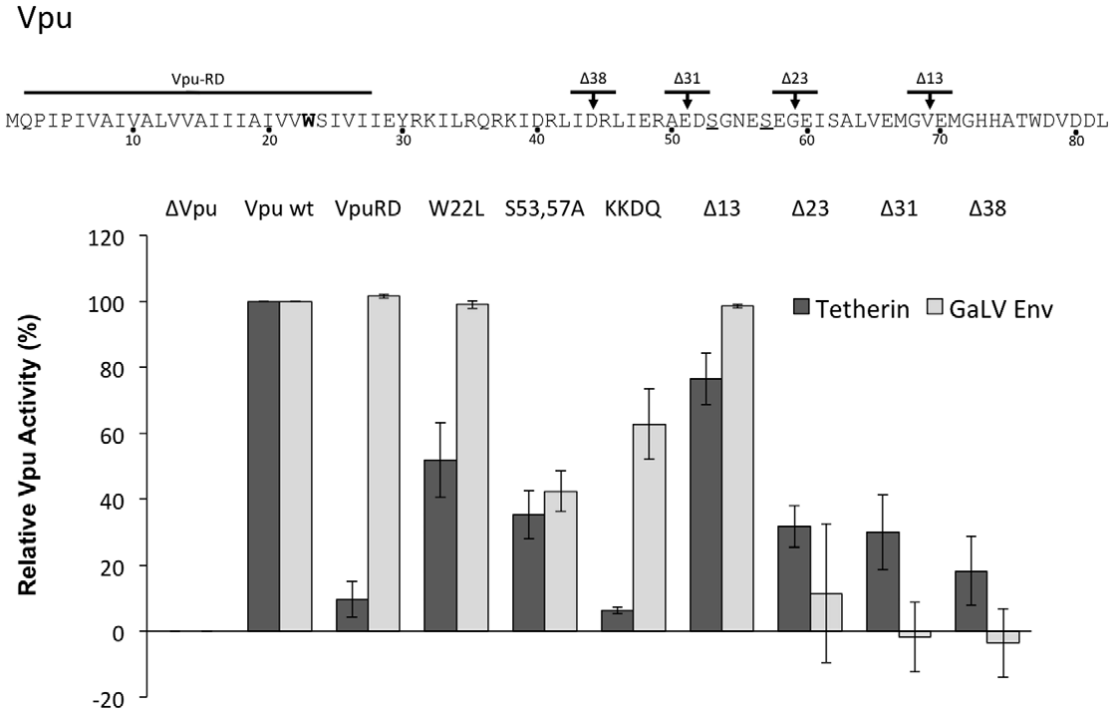
Features required for Vpu-mediated antagonism of targets, tetherin (dark bars) and GaLV Env (light bars). (Top) Location of VpuRD, W22L (bold), critical serines 53,57 (underline) and truncations (arrows) are noted in the Vpu schematic. (Bottom) Relative Vpu activity is shown as mean averages (n = 3–4, ±SE) calculated by normalizing infectious units per ml for each mutant Vpu relative to Vpu wildtype (Vpu wt) (100%) and no Vpu (ΔVpu) (0%).

Previous studies have demonstrated that Vpu's transmembrane domain (TMD) and cytoplasmic tail (CT) promote tetherin antagonism while only the Vpu CT has been identified for GaLV Env restriction [2], [3], [24], [43]. VpuRD, a transmembrane “scrambled” mutant, is known to fully restrict CD4, but is ineffectual against tetherin [24]. However, there have been conflicting reports about the importance of the TMD in CD4 restriction [25], with some studies suggesting a role of a conserved tryptophan (W22) in the C-terminal region [20], [21]. We therefore sought to further investigate the role of Vpu's TMD by employing two previously described TMD mutants: VpuRD and W22L [16], [20], [24]. We introduced both of these mutants into our proviral system and tested their activity against tetherin and GaLV Env (Figure 2). As previously reported, both VpuRD and W22 mutants had decreased activity against tetherin [43]–[45]. However, both mutants exhibited wildtype activity against GaLV Env. In addition, we also included serine to alanine mutations at positions 53, 57. These serines are highly conserved and have been previously reported to be essential in tetherin and CD4 downmodulation [43], [46]. As expected, the serines are important in downmodulation of both tetherin and GaLV Env, presumably through their ability to mediate β-TrCP activity.

### Vpu localization restricts antagonism of tetherin and GaLV Env

The subcellular location where CD4 and tetherin are targeted appears to be distinct. While action against CD4 has been reported to be exclusively in the RER, action against tetherin is generally believed to occur in a post-ER compartment [18], [23], [31], [47], [48]. Previous studies demonstrated that Vpu retention in the RER by the addition of a putative retrieval motif prevents downmodulation of tetherin at the PM [44], [47]. We found that placement of the KKDQ ER-retention motif on the C-terminus of Vpu, exactly as previously described [47], reduced its ability to restrict either target, though the effect on tetherin restriction was more severe. These data are consistent with direct or indirect interactions between Vpu and both target proteins in a post-ER region.

### Conserved amino acid features in Vpu cytoplasmic tail are required for activity

**Figure 3:**
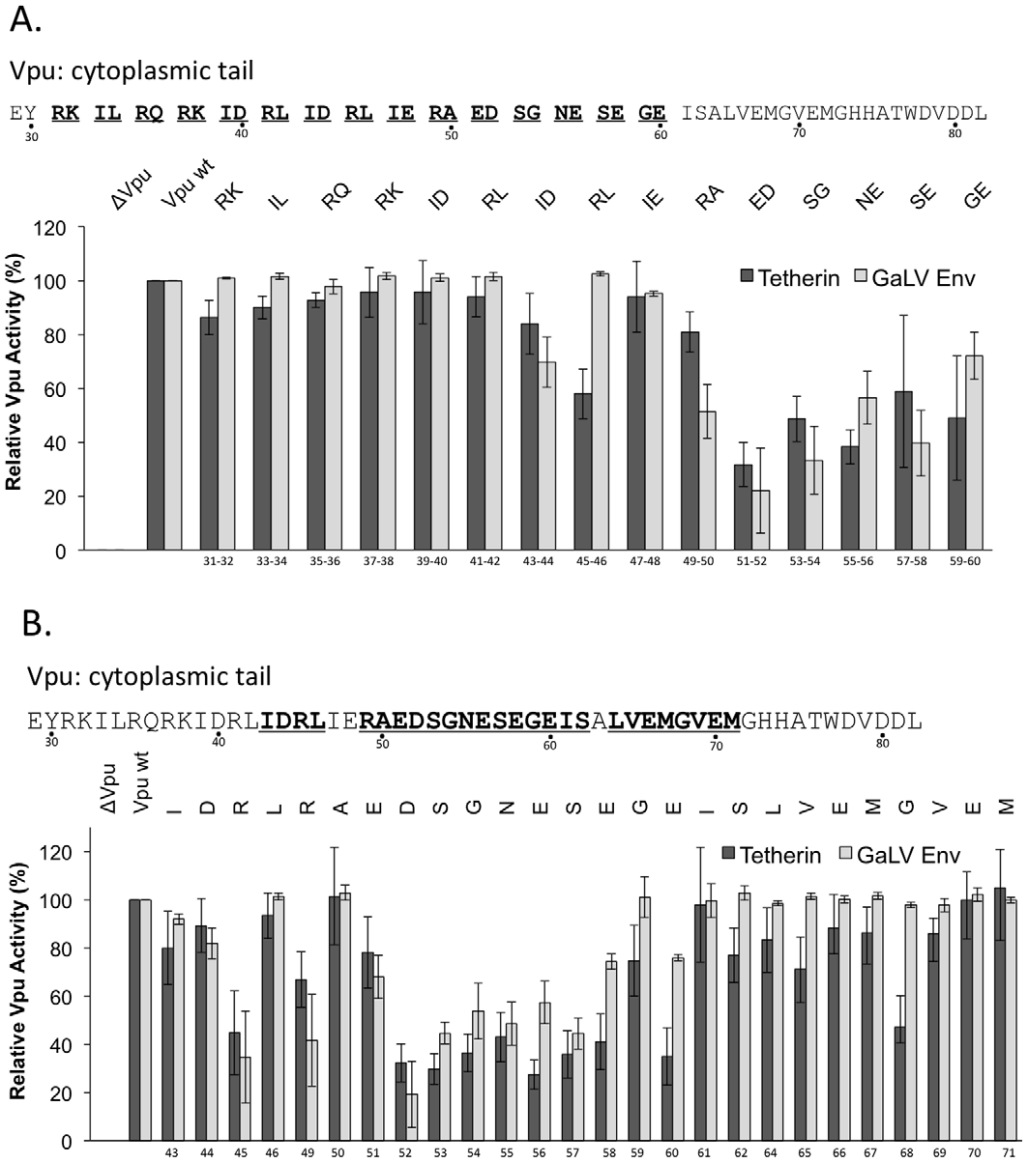
Alanine mutagenic scan of Vpu reveals antagonistic regions for downmodulation of tetherin (dark bars) and GaLV Env (light bars). Amino acids were mutated to alanine, with the exception of alanine, which was mutated to serine (A) A double alanine-mutagenic scan was performed on the cytoplasmic tail region of Vpu (double mutations, underlined). (B) An individual amino acid alanine scan was analyzed for amino acids identified in the double-alanine scan (bold, underlined) and relative Vpu activity was measured. Relative Vpu activity is shown as mean averages (n = 3–4, ±SE) calculated by normalizing infectious units per ml for each mutant Vpu relative to Vpu wildtype (Vpu wt) (100%) and no Vpu (ΔVpu) (0%).

Next, we generated truncation mutations in Vpu to determine the minimal sequence required for modulation of the two targets in this system. For both tetherin and GaLV Env, truncation beyond 13 C-terminal amino acids (Δ13) resulted in a decrease in Vpu function, although for tetherin this decrease was progressive (Figure 2). To identify critical regions upstream of Δ13, we mutated two residues at a time to alanine and assayed for activity. For both targets, Vpu was most sensitive to mutations within the conserved hinge region while upstream regions were less sensitive (Figure 3A). Unlike reported findings for BH10 Vpu R30A,K31A [48], mutations located within the YRKIL trafficking motif, we did not observe a decrease in infectivity in the presence of tetherin with our HXB2 Vpu system. Although both are subtype B and almost identical in amino acid sequence, we cannot exclude that subtle variation between the two strains may explain observed differences.

We then sought to identify specific amino acids required in the CT by scanning individual point mutants through substitution of alanine for individual amino acids, with the exception of alanine which was substituted with serine. Interestingly, almost all amino acids within the Vpu-hinge region between amino acids 51–60, not solely the serines 53, 57, were sensitive to disruption (Figure 3B). A recent report suggested that a putative trafficking motif, ExxxLV, located between residues 60–65 is required for tetherin antagonism [49]. In agreement with this report, we found that E60A disrupted tetherin activity, but the effects of L64A, and V65A alone were more modest. These results demonstrate Vpu's requirement for conservation of the hinge region for antagonism of two distinct protein targets. Because alanine substitution should not affect physical accessibility of the hinge region by proteins such as β-TrCP, we presume that modification of the conserved features, such as the acidic amino acids, disrupts recognition of Vpu by cellular factors or Vpu's ability to interact with targets.

## Discussion

Here we have identified shared critical features in Vpu required for restriction of two distinct proteins, tetherin and the glycoprotein GaLV Env. With the exception of the TMD region, Vpu requires similar features to counteract both targets. Our Vpu screen raises the question: why are similar features in Vpu required for modulation of two disparate target proteins? We propose that Vpu utilizes multiple regions for three somewhat overlapping steps in both restriction pathways: i) retention through interaction, ii) modification and redirection, and iii) degradation. In the case of tetherin, interaction occurs between the TMDs and for CD4 interaction occurs in the CTs and is absolutely required for antagonism [24], [34], [44], [50], [51]. The importance of TMD interactions is highly evident in the evolution of species and subtype specificity of Vpu antagonism of tetherin [47], [52], [53]. In the second step, we postulate that Vpu's CT-hinge region is required for both tetherin and GaLV Env modification and redirection. The hinge region likely represents a collective β-TrCP recognition motif, with serines housed within a conserved acidic stretch of amino acids. How Vpu modifies and subsequently redirects targets is not yet fully understood, although emerging data suggests a role of ubiquitination of both tetherin and CD4. While CD4 is polyubiquitinated, it is currently unclear whether tetherin is multiply monoubiquitinated or polyubiquitinated, hallmarks of redirection for lysosomal or proteasomal degradation, respectively [18], [27], [54], [55]. In the final step of restriction, degradation of targets may occur. CD4 is directed for degradation through ERAD-proteasomal targeting [5], [6], [14]–[17], However, the role of degradation for tetherin is unclear, with some data suggesting lysosomal [33], [36], [55] or proteasomal degradation [37]. Interestingly, although tetherin restriction can occur independently of the degradation, possibly through retention-based interactions, recent work demonstrates a significant role for lysosomal degradation of newly synthesized tetherin [29]. We suspect that degradation may represent a late stage in restriction and may not be required until available Vpu becomes saturated.

Through our systematic alanine mutagenic library of the Vpu cytoplasmic tail, we identified specific amino acids contributing to the antagonism of two distinct targets, tetherin and a viral glycoprotein, GaLV Env. Interestingly, we demonstrated a role for multiple amino acids within the CT hinge region and the importance of Vpu localization in restriction. Altogether our findings, along with other mutagenic Vpu studies, suggest that Vpu has unique regions mediating interaction with targets, while it uses conserved features within the CT to ultimately redirect and potentially degrade target proteins.
